# Poly(ADP-ribose) polymerase (PARP) inhibitors in cardiovascular, and cerebrovascular diseases: mechanisms, current trends and challenge for clinical translation

**DOI:** 10.7717/peerj.20842

**Published:** 2026-02-20

**Authors:** Jinlin Fan, Yanfangfei Song

**Affiliations:** Shengjing Hospital of China Medical University, China Medical University, Shenyang, China

**Keywords:** PARP1, Cardiovascular diseases, Cerebrovascular diseases, PARP inhibitor

## Abstract

Poly(ADP-ribose) polymerase (PARP) inhibitors constitute the inaugural targeted therapy shown to enhance the prognosis of individuals with hereditary tumors, initially utilized in the management of patients with germline *BRCA1/2-*associated breast cancer. With ongoing research, PARP inhibitors (PARPi) are currently under extensive investigation for their applicability across a spectrum of diseases, encompassing oncology, cardiovascular diseases, and cerebrovascular diseases. This narrative review provides a comprehensive synthesis of the biological rationale, existing evidence, recent advancements, and prospective future directions of PARPi in the treatment of cancers, cardiovascular diseases, and cerebrovascular disorders. We provide a comprehensive overview of the recent advancements, advantages, and limitations associated with both clinically approved and investigational PARPi. Beyond their application in oncology, PARPi demonstrate significant potential in other therapeutic domains, including cardiovascular diseases. As our comprehension of the biological functions of PARP and its molecular mechanisms advances, it is anticipated that the therapeutic applications of these inhibitors will broaden considerably. Future research endeavors should prioritize the identification of predictive biomarkers across various diseases and the development of strategies to circumvent drug resistance. Consequently, the integration of fundamental and clinical research on PARPi across diverse diseases is essential to establish a foundational framework for clinical translation.

## Introduction

Poly(ADP-ribose) polymerases (PARPs) constitute a family of nuclear proteins comprising 17 members that perform critical cellular functions. Their core mechanism involves PARylation (poly-ADP-ribosylation), through which they auto-modify and modify DNA damage response (DDR) proteins. This post-translational modification enables PARPs to orchestrate essential biological processes including DNA repair, gene transcription regulation, and modulation of cell death pathways (Huang & Kraus, 2022). Under physiological conditions, PARPs are the critical mediator for maintaining cellular homeostasis. They primarily localize within the nucleus and maintains tight chromatin association, actively participating in diverse biological processes including DDR (recognizing and repairing both single-strand breaks (SSBs) and double-strand breaks (DSBs)), chromosomal stability maintenance, transcriptional regulation, and cellular proliferation and differentiation (Morales et al., 2014; Rodríguez et al., 2015; Verdone et al., 2015; Xu et al., 2014). PARP1 is the predominant isoform of the PARPs family, with PARP1 accounting for 85–90% of total cellular PARylation activity (Xu et al., 2014). PARP1 and PARP2 both participate in DNA repair, however, PARP2 lacks the N-terminal DNA-binding domain which present in PARP1. Additionally, PARP2 regulates epigenetic, proliferative, and inflammatory processes. Notably, some PARP inhibitors (PARPi) targeting PARP1 also exhibit inhibitory effects against PARP2 (Liu, Pillai & Leung, 2025).

PARP1 exhibits a dual role in response to DNA damage. Under moderate DNA damage, PARP1 acts as an early sensor of DNA lesions by rapidly binding to the damaged site and synthesizing PAR chains using NAD^+^. As these PAR chains accumulate on PARP1 (PARylation), their increasing negative charges generate electrostatic repulsion with the negatively charged DNA. This repulsion causes PARP1 to detach from the damage site, thereby exposing the DNA break and allowing downstream repair proteins to be efficiently recruited and complete base excision repair. This sophisticated mechanism promotes cellular repair and survival. However, under severe genomic damage, PARP1 becomes excessively activated and triggers excessive PAR synthesis, thus leading to rapid depletion of intracellular NAD^+^ and ATP. As the storage of NAD^+^ and ATP is exhausted, the inability to complete auto-PARylation–mediated release prevents the recruitment of DNA repair proteins, leading to reactive oxygen species (ROS) overproduction, mitochondrial collapse, and failure of DNA repair. Collectively, these events highlight that under severe genomic injuries, PARP1 activation exhibits a dual role, switching from a protective DNA repair mechanism to a pro-death signal under sustained stress (Wen, Yin & Garg, 2018).

Currently, the primary clinical application of PARPi is in anticancer therapy. By competitively binding to the NAD^+^ site on PARP enzymes, PARPi blocks the PARylation, thereby impeding efficient DDR. In cancer with Homologous Recombination Deficiency (HRD) mutations, the inhibition leads to the persistent accumulation of DSBs, ultimately inducing selective cell death through synthetic lethality mechanism (Jiang et al., 2019; Mittica et al., 2018; Thomas, Murai & Pommier, 2018). Furthermore, pharmacological studies have demonstrated that PARPi significantly potentiates the cytotoxic effects of various conventional therapies. These include ionizing radiation and DNA-damaging agents, and topoisomerase I inhibitors, thus enhancing the chemosensitivity of tumors (Bowman et al., 2001; Cimmino et al., 2007; Griffin et al., 1995; Tentori & Graziani, 2005; Tentori, Portarena & Graziani, 2002).

Beyond the success of PARPi in oncology, the therapeutic spectrum of PARPi has expanded to encompass a variety of cardiovascular and cerebrovascular diseases, especially in ischemia-reperfusion and inflammatory diseases. Studies have indicated the therapeutic potential of PARPi in pathological models of hemorrhagic shock (Lord et al., 2009), cardiovascular diseases like myocardial infarction (Abdallah et al., 2007; Gerö et al., 2014; Huang et al., 2011; Pacher et al., 2002), pulmonary diseases (Genovese et al., 2005; Meloche et al., 2014), cerebrovascular diseases like ischemic stroke (Cai et al., 2024; Salman et al., 2025). Unfortunately, despite extensive studies on PARPi in oncology, few studies have explored the clinical application of PARPi in the cardiovascular and cerebrovascular fields (Ke et al., 2019; Kim et al., 2025), and their roles in cardiovascular and cerebrovascular diseases remain fragmented and understudied. Existing reviews either focus narrowly on oxidative stress or discuss PARP biology without integrating metabolic, inflammatory, and cell-death mechanisms across disease types. Moreover, no current review systematically links PARP-driven pathological pathways with preclinical evidence and emerging therapeutic strategies. To address these gaps, this review summarizes the current clinical applications of PARPi in oncology and major focus on PARPi’s preclinical research progress and future clinical potential in cardiovascular and cerebrovascular diseases. These insights are particularly relevant for preclinical oncologists designing biomarker-driven treatment strategies, pharmaceutical scientists developing next-generation PARP inhibitors and combination therapies, and basic research scientists investigating the fundamental principles of genomic instability and DNA repair.

## Search methodology

This review analyzed relevant literature published between 2000 and 2025, retrieved from PubMed and Web of Science. A comprehensive literature search was performed using a strategy that combined subject headings (*e.g*., MeSH) and free-text words. Search terms were related to two main concepts: (1) PARP inhibition and (2) cardiovascular or cerebrovascular diseases. Terms for the first concept included “PARP inhibitors”, “PARPi”, “Poly(ADP-ribose) polymerase inhibitors”, and names of specific drugs (*e.g*., “Olaparib”, “Niraparib”). For the second concept, terms for cardiovascular diseases included “cardiovascular” “myocardial infarction”, “heart failure”, “atherosclerosis”, “cardiac ischemia”, and “endothelial dysfunction”. Terms for cerebrovascular diseases included “cerebrovascular diseases” “stroke”, “cerebral ischemia”, “hemorrhagic stroke”, and “neuroinflammation”. The retrieved articles were screened by title, followed by abstract and keyword evaluation. Subsequently, the full texts of potentially relevant articles were retrieved for a final eligibility assessment.

## Molecular mechanisms of parpi in cancer

### The role of PARPs in DNA damage response

Genomic integrity is constantly challenged by DNA lesions, such as SSBs and DSBs (Lord & Ashworth, 2012). The PARP family, particularly PARP1, serves as a primary sensor for SSBs. Upon detecting a break, PARP1 binds to the damaged DNA, triggering its catalytic activity. Utilizing NAD^+^ as a substrate, PARP1 synthesizes long chains of PAR onto itself and other acceptor proteins, a process known as PARylation. This signaling cascade recruits a scaffold of DNA repair proteins, including X-ray Repair Cross-Complementing Protein 1 (XRCC1) (Javle & Curtin, 2011), to the lesion site, facilitating high-fidelity SSB repair and maintaining genomic stability.

### Synthetic lethality: the core therapeutic principle

The therapeutic efficacy of PARPi is primarily rooted in the concept of “Synthetic Lethality.” This occurs when the simultaneous loss of two separate gene functions leads to cell death, whereas the loss of either one alone is compatible with cell viability. The classic example involves the interplay between PARP-mediated SSB repair and the Homologous Recombination (HR) pathway for DSB repair.

PARP1 predominantly orchestrates SSBs repair. Unrepaired SSBs persisting through DNA replication phase may escalate into more deleterious DSBs. Two principal pathways mediate DSB resolution: error-prone non-homologous end joining (NHEJ) and high-fidelity HR. HR operates predominantly during S/G2 phases, utilizing intact sister chromatids as repair templates to ensure genomic accuracy. Critical to this process is the BRCA1-Associated RING Domain Protein 1-BRCA1 C-Terminal (BARD1-BRCT) domain’s interaction with PAR motifs, which facilitates BRCA1’s early recruitment to DNA lesion sites (Li & Yu, 2013). The BARD1/BRCA1 heterodimer plays a pivotal role in HR-mediated repair of DSBs (Hu et al., 2014). This complex exhibits intrinsic ubiquitin ligase activity, specifically targeting components of the RNA polymerase II (Pol II) complex for proteasomal degradation. This degradation process removes transcription-replication conflict barriers, thereby facilitating HR initiation (Kleiman et al., 2005; Wright, Shah & Heyer, 2018). When severe DSBs persist due to HR deficiency, the failure to resolve these lesions ultimately triggers programmed cell death.

Homologous recombination deficiency (HRD), arising from mutations, deletions, or epigenetic silencing of genes within the HR repair pathway such as *BRCA1/2* (Harvey-Jones et al., 2024), *PALB2* (Wang et al., 2025b), and *RAD51* (Hu et al., 2023) impairs the capacity of cell for accurate DSB repair and elevates the risk of tumorigenesis. PARP1 acts as a critical mediator for the survival of various malignancies, owing to its pivotal role in DDR. Consequently, cancer cells, particularly those with inherent defects in DNA repair pathways, become highly dependent on PARP1 activity to repair DNA damage and sustain their oncogenic proliferation (Yang et al., 2025b). HRD provides the rationale for a therapeutic strategy targeting PARP1, a concept known as synthetic lethality. In HR-deficient cells, the inhibition of PARP1/2 by PARPi leads to the accumulation of DSBs. These HR-deficient cells are incapable of effectively resolving such lesions, and HR-deficient cancer cells exhibit synthetic lethality through repair pathway collapse (reliance on error-prone alternative NHEJ increases genomic instability) and replication catastrophe (persistent single-ended Double-Strand Breaks (seDSBs) overwhelm residual repair capacity) (Liu et al., 2014a). ultimately leading to cell death. This synthetic lethality paradigm explains the clinical efficacy of PARPi in HR-deficient cancers (Bryant et al., 2005; Dedes et al., 2011; Eppink et al., 2012; Farmer et al., 2005; Helleday, 2011; Javle & Curtin, 2011; Krawczyk et al., 2011; Sousa et al., 2012). Beyond SSB repair blockade, PARPi also impair backup-NHEJ pathways, further compromising DSB repair fidelity (Audebert, Salles & Calsou, 2004; Wang et al., 2006). In contrast, normal cells, which possess a proficient HR pathway, can tolerate the loss of PARP activity and are therefore significantly less affected (Bryant et al., 2005; Illuzzi et al., 2014; Xiong et al., 2025), accounting for the selective cytotoxicity of PARPi.

### PARP trapping: a key determinant of cytotoxicity

**Figure 1:**
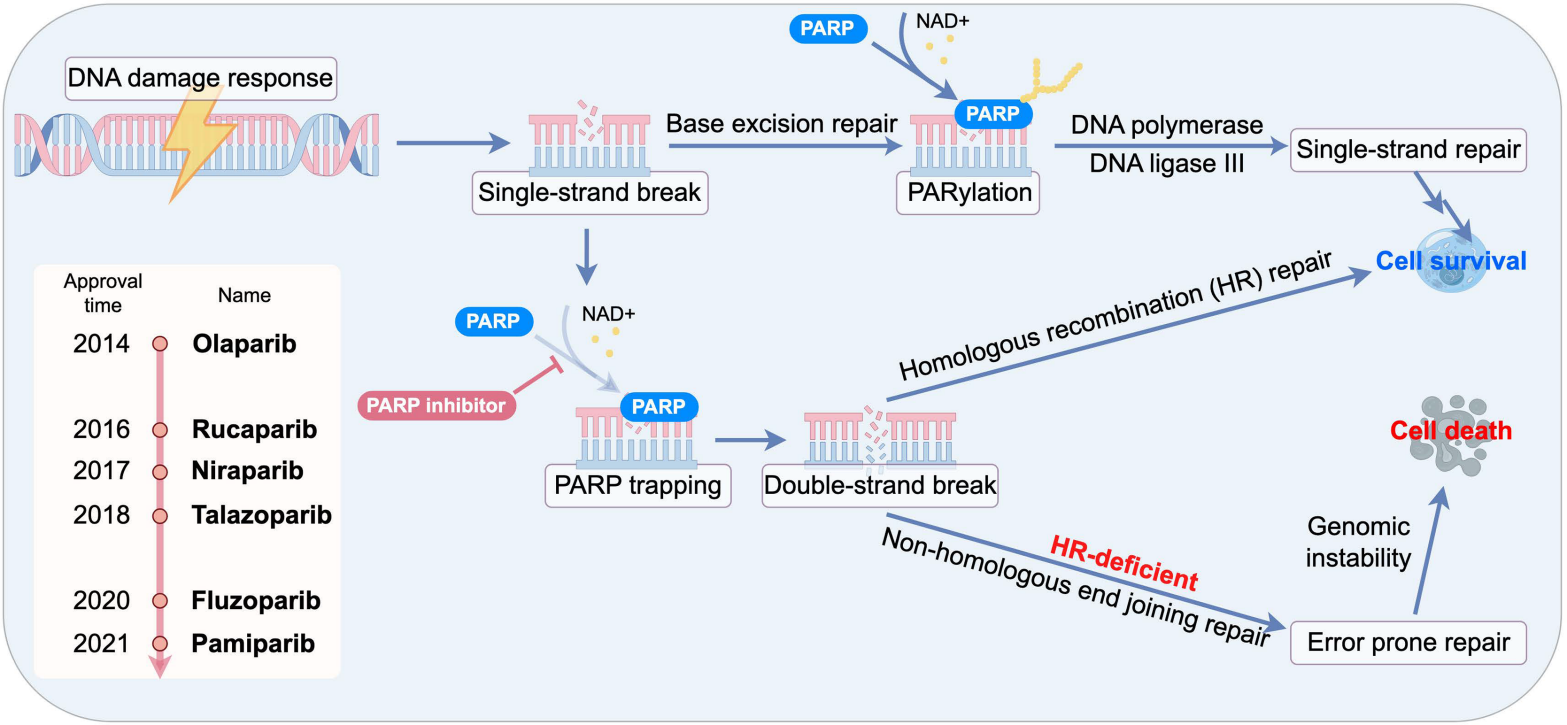
Schematic of synthetic lethality and PARP trapping.

Beyond enzymatic inhibition, the most potent PARPi exert their effect through a mechanism known as “PARP trapping”. These inhibitors bind to the NAD^+^ pocket of PARP1, locking the enzyme onto the DNA at the site of damage. This creates a cytotoxic PARP-DNA complex, which is a physical obstacle to DNA replication and transcription machinery. The formation of these trapped complexes, rather than the mere inhibition of PARylation, is now considered the primary driver of PARPi-induced cytotoxicity and is particularly lethal to rapidly proliferating cancer cells. The trapped PARP1-DNA complexes create physical barriers to replication fork progression. Crucially, rapidly proliferating cancer cells exhibit heightened vulnerability to these replication-associated lesions due to their elevated replication stress, resulting in selective lethality through mitotic catastrophe. This mechanistic distinction explains the preferential cytotoxicity toward malignant cells over normal counterparts (Murai et al., 2012; Pommier, O’Connor & de Bono, 2016). The PARP trapping effect occurs independently of PARylation activity and is modulated by specific mutations within the catalytic domain, which may extend the persistence of DNA damage–induced foci (Pettitt et al., 2018). Notably, the potency of different PARPi directly correlates with their ability to trap PARP1 on DNA (Fig. 1).

Collectively, these mechanistic insights establish PARP inhibition as a powerful therapeutic strategy for cancers characterized by high genomic instability. By blocking PARP-dependent single-strand break repair, exploiting synthetic lethality in homologous recombination–deficient tumors, and inducing cytotoxic PARP–DNA trapping, PARPi effectively convert endogenous DNA lesions into lethal replication stress. Together, these mechanisms provide a compelling biological rationale for the broad clinical implementation of PARP inhibitors in oncology.

However, the biological functions of PARPs extend well beyond tumor cells and DNA repair. Their central involvement in in metabolic regulation, oxidative stress responses, inflammation, and cell survival pathways suggests that PARP modulation may hold significant therapeutic promise for non-cancer diseases. Building upon the mechanistic principles elucidated in cancer research, emerging evidence highlights a broader translational potential of PARPi in cardiovascular and cerebrovascular diseases.

## Therapeutic potential of parpi in cardiovascular and cerebrovascular diseases

### Divergent therapeutic paradigm: from cytotoxicity to cytoprotection

The application of PARPi in cardiovascular and cerebrovascular diseases operates on a fundamentally different principle, while the efficacy of PARPi in oncology is rooted in inducing synthetic lethality and cytotoxic PARP trapping in rapidly proliferating cancer cells (Wang et al., 2025a). In oncological diseases, the rapid proliferation of tumor cells induces substantial DNA damage, and the pre-existing high levels of PARylation in cancer enable PARPi to exert therapeutic effects by suppressing PARP1-mediated DNA damage repair. Unlike their cytotoxic roles in oncology, PARPi function through distinct non-proliferative mechanisms in cardiovascular and cerebrovascular diseases, where mature cardiomyocytes and neurons are terminally differentiated and possess minimal regenerative capacity.

The therapeutic goal is not to kill these cells, but to protect them from acute injury and chronic stress. The primary mechanism of action for PARPi in this context shifts from targeted cytotoxicity to cyto-protection, focused on mitigating the downstream consequences of PARP1 hyperactivation triggered by oxidative stress, inflammation, and ischemia.

### Pathophysiological mechanisms of PARP1 in cardiovascular and cerebrovascular diseases

**Figure 2:**
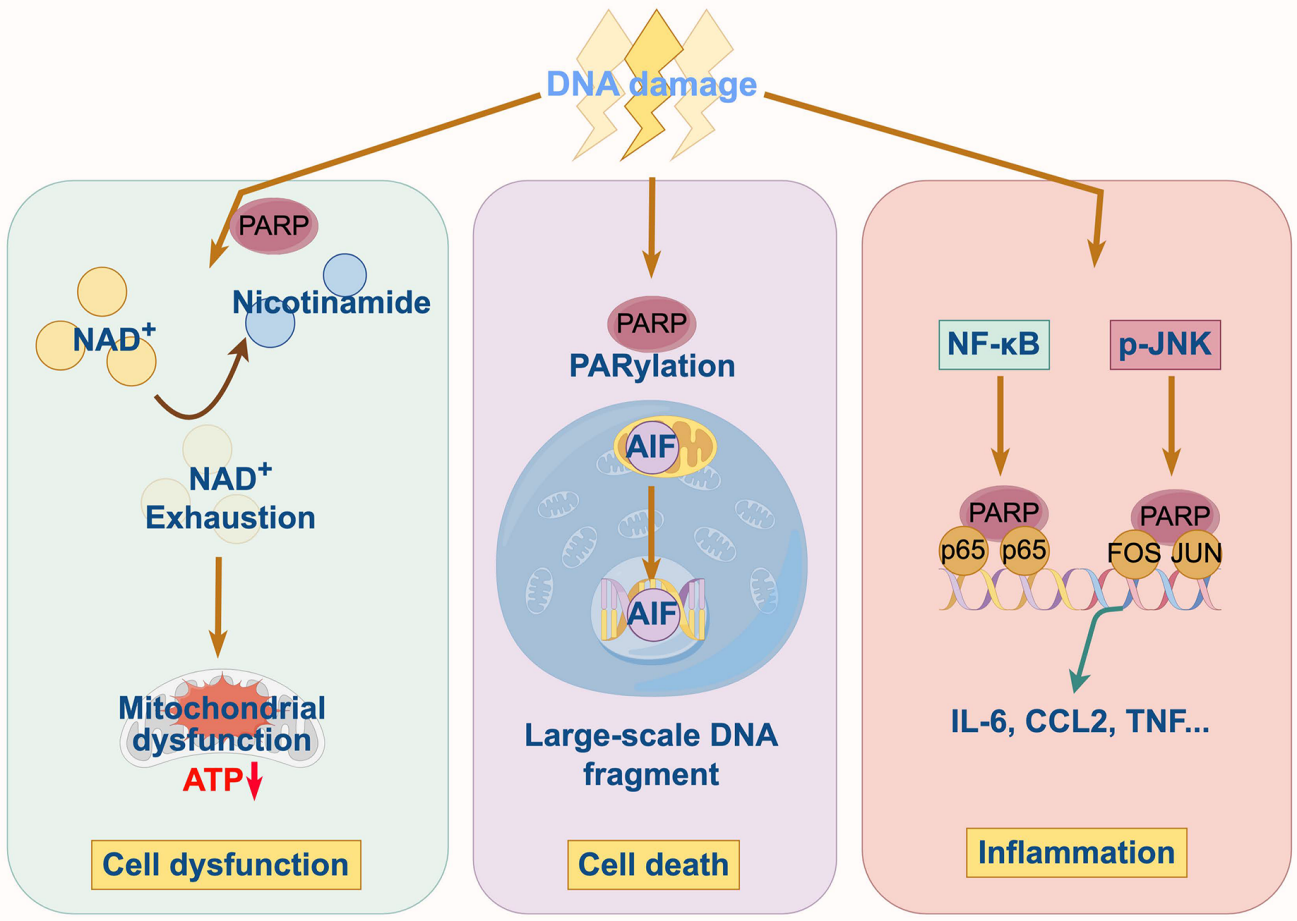
Pathophysiological mechanisms of PARP1 in cardiovascular and cerebrovascular diseases.

In cardiovascular and cerebrovascular diseases, a common feature is the overproduction of reactive oxygen and nitrogen species (ROS/RNS), which inflict significant DNA damage. This persistent DNA damage triggers the hyperactivation of PARP1, initiating a vicious cycle of cellular injury through three interconnected mechanisms as follow (Fig. 2).

#### Energy depletion and metabolic collapse

PARP1 hyperactivation consumes vast quantities of cellular NAD^+^, a critical coenzyme for energy metabolism. The catastrophic depletion of the NAD^+^ pool cripple glycolysis and oxidative phosphorylation, leading to a severe drop in ATP levels. This energy crisis impairs essential cellular functions, particularly in high-energy-demand cells like cardiomyocytes and neurons, ultimately driving them towards programmed cell death.

In hypertension or heart failure, mitochondria also serve as primary sources of endogenous ROS generation (Ordog et al., 2021). When the antioxidant defense system becomes insufficient, endogenous reactive oxygen species (ROS) accumulate and damage key cellular structures, including nuclear and mitochondrial DNA (Kowaltowski et al., 2009). ROS directly oxidize nucleobases (Shokolenko et al., 2009; Srinivas et al., 2019), thereby activating PARP1 and triggering severe depletion of mitochondrial NAD^+^ levels, which exacerbates oxidative protein and DNA damage. Endothelial dysfunction in diabetes, hypertension, atherosclerosis, shock, and heart failure is mechanistically linked to localized overproduction of reactive oxygen/nitrogen species and PARP activation within vascular endothelial cells. Myocardial or endothelial cells with DNA damage can enter three primary pathways depending on the severity of DNA lesions and the extent of PARP activation (Pacher & Szabó, 2007). Under mild-to-moderate DNA damage, such as occurs in cardiomyocytes or endothelial cells during transient ischemia, PARP1 activation recruits DNA repair enzymes to correct lesions, promoting cellular survival. Moderate PARP activation reduces cellular NAD^+^ levels without causing lethality; such energy-compromised cells exhibit reversible dysfunction, and PARPi can partially restore energy metabolism to rescue cells from this dysfunctional state (Graziani & Szabó, 2005; Pacher & Szabó, 2007; Virág et al., 2013). The third pathway, triggered by extensive DNA damage, involves PARP hyperactivation leading to catastrophic NAD^+^ depletion and mitochondrial collapse. In this pathway, PARPi show limited efficacy by partially attenuating mitochondrial dysfunction, ROS release, and mitochondrial death factor liberation (Hong, Dawson & Dawson, 2004; Wang, Dawson & Dawson, 2009). Activation of the DNA damage-PARP1-NAD^+^ axis compromises genomic integrity, disrupts mitochondrial homeostasis, and ultimately culminates in cardiomyocyte dysfunction (Hassa & Hottiger, 2008). This exacerbates ROS production, elevates intracellular Ca^2+^, disrupts mitochondrial membrane integrity *via* permeability transition, and ultimately induces necrosis in cardiomyocytes and endothelial cells due to impaired glycolysis, Krebs cycle dysfunction, and mitochondrial electron transport chain failure (Dawson & Dawson, 2004; Halmosi et al., 2001; Song et al., 2013; Virág & Szabó, 2002; Wang, Dawson & Dawson, 2009; Yu et al., 2006). This axis also operates in coronary artery disease, where hypoxia-reoxygenation cycles exacerbate oxidative stress and free radical release, promoting DNA damage, PARP activation *via* PARylation stimulation, and consequent NAD^+^/ATP depletion (Ramos & Brundel, 2020). Furthermore, the DNA damage-PARP1-NAD^+^ axis contributes to hypertension-associated endothelial dysfunction (Carrillo et al., 2005), Lamin A/C gene (LMNA) mutation-induced dilated cardiomyopathy (Diguet et al., 2018), and peripartum cardiomyopathy pathogenesis (Ricke-Hoch, Pfeffer & Hilfiker-Kleiner, 2020).

In summary, PARP1 hyperactivation serves as a central pathogenic hub that links oxidative stress, DNA damage, and metabolic collapse. Cells with high energetic demands—such as cardiomyocytes, endothelial cells, and neurons—are particularly vulnerable to NAD^+^ depletion and the downstream consequences of excessive PARP1 activation. Optimizing PARPi dosing, therapeutic timing, and disease-stage specificity is therefore critical in conditions such as hypertension, diabetes, ischemia–reperfusion injury, and heart failure. While PARPi can restore metabolic homeostasis under moderate DNA damage, their therapeutic efficacy becomes markedly constrained once severe genomic injury, catastrophic NAD^+^/ATP depletion, or mitochondrial permeability transition has occurred. Consequently, combination approaches—such as co-administration of PARPi with NAD^+^ precursors, mitochondrial stabilizers, or ROS scavengers—may offer superior cytoprotective benefits. Furthermore, the development of tissue-targeted delivery systems that preferentially direct PARPi to cardiac or vascular tissues could help minimize systemic metabolic liabilities. Collectively, these strategies hold promise for achieving therapeutic benefit without compromising the essential DNA repair functions of PARP1.

#### Modulation of cell death pathways

The hyperactivation of PARP1 is a critical driver of cell death. Cellular energy status is a critical determinant of the mode of cell death. The severe ATP depletion caused by PARP1 hyperactivation inhibits the energy-dependent apoptotic program and forces the cell into an uncontrolled, pro-inflammatory necrotic pathway. By preserving NAD^+^ and ATP pools, PARPi can reprogram cell fate, shifting the balance from necrosis towards apoptosis or even promoting cell survival and functional recovery.

Research has demonstrated that pharmacological inhibition of PARP1 shifts cell death modalities to apoptosis (Virág, Salzman & Szabó, 1998). In cells subjected to ROS damage, PARPi prevents necrotic cell death while permitting apoptotic pathways or even restoring normal phenotypic states, thereby reprogramming cell fate from necrosis to apoptosis or phenotypic normalization. Notably, PARPi or genetic ablation provides the most profound protection in disease models dominated by cell death, including stroke, myocardial infarction, and mesenteric ischemia-reperfusion injury (Kim et al., 2018; Virág & Szabó, 2002).

Cell death is also further promoted by the mitochondrial release of apoptosis-inducing factor (AIF) (Wang et al., 2016). Persistent PARP1 activity also suppresses NAD^+^-dependent deacetylase Sirtuin 1 (SIRT1) and AMP-activated protein kinase (AMPK), leading to mitochondrial dysfunction. Conversely, PARPi counteract these effects by preserving NAD^+^ levels, which in turn activates the SIRT1-AMPK pathway, restores mitochondrial function, and promotes cell survival (Cheng et al., 2003). They also maintain cellular homeostasis by preventing mitochondria-mediated cell death through the inhibition of AIF release. In the context of hyperglycemia, PARP1 activation directly modulates apoptotic signaling. Pharmacological inhibition of PARP1mitigates high glucose-induced cardiomyocyte apoptosis by downregulating cleaved-Caspases3 and cleaved-Caspases9 and concurrently activating the pro-survival insulin-like growth factor 1 receptor (IGF-1R)/Akt pathway (Qin et al., 2016).

Ferroptosis is a distinct form of regulated cell death driven by the iron-dependent accumulation of lipid peroxides and ROS, distinguishing it from canonical pathways such as apoptosis, necroptosis, and autophagy (Dixon et al., 2012). Recent research has revealed that PARPi can mitigate ferroptosis in cardiomyocytes, vascular smooth muscle cells (VSMC), and leukocytes (Yang et al., 2025a). PARP1-mediated PARylation-dependent ubiquitination of DNA Polymerase gamma (POLG) in VSMC ferroptosis (Yang et al., 2025a). The other research found that the protective effect of PARPi may be mediated by PARP1 ability to promote mitophagy, which preserves mitochondrial integrity and prevents the triggers of ferroptosis (Liu et al., 2024). Collectively, these findings indicate a potent protective role for PARPi in programmed cell death pathways.

#### Pro-inflammatory signaling

PARP1 functions as a transcriptional regulator that influences inflammatory responses and cell survival through the modulation of nuclear factor-κB (NF-κB), p53, and hypoxia-inducible factor-1α (HIF-1α) (Elser et al., 2008; Hassa et al., 2003; Salameh et al., 2020; Wiman, 2013). Its activation promotes the expression of cytokines, chemokines, and adhesion molecules, fueling a persistent inflammatory state that contributes to endothelial dysfunction, atherosclerotic plaque progression, and adverse tissue remodeling. In the myocardial ischemia-reperfusion injury, PARP1 hyperactivation amplifies NF-κB signaling, which upregulates adhesion molecules like P-selectin and intercellular adhesion molecule-1 (ICAM-1) and enhances leukocyte-endothelial interactions (Song et al., 2013; Zingarelli, Salzman & Szabó, 1998). This cascade promotes neutrophil infiltration into myocardial tissue, thereby exacerbating inflammation and tissue injury. Consistent with this role, treatment with PARPi has been shown to decrease the levels of inflammatory mediators, including tumor necrosis factor-α (TNF-α) leading to the attenuation of myocardial inflammation (Yang, Zingarelli & Szabó, 2000). SIRT1, a member of Sirtuin family, exerts multifaceted endothelial protection by deacetylating substrates that normally suppress NF-κB-mediated gene expression. However, PARP1 inhibits SIRT1 activity in cardiomyocytes, and during severe ischemia, this imbalance accelerates caspase activation and PARP cleavage prior to atherosclerotic plaque rupture and necroptosis (Alhosaini et al., 2021; Kauppinen et al., 2013; Xu et al., 2014; Yeung et al., 2004).

Beyond its canonical role in DNA repair, PARP1 acts as a transcriptional integrator that connects metabolic stress to inflammatory gene expression *via* regulators such as NF-κB and HIF-1α. In cardiovascular and cerebrovascular contexts, PARP1 coordinates a multifaceted network of metabolic and inflammatory signals. Elucidating its context-specific functions and optimizing targeted intervention strategies will be key to realizing the full translational potential of PARP1 inhibition in clinical medicine.

### Preclinical evidence in pathological of cardiovascular and cerebrovascular diseases

#### Ischemic diseases

In ischemic diseases, ischemia-reperfusion (I/R) injury primarily induces cardiomyocyte and neuronal cell death through programmed cell death mechanisms, with apoptosis playing a significant contributory role. I/R-triggered ROS generation causes oxidative DNA strand breaks, thereby activating PARP1. PARP activation disrupts cellular metabolic efficiency by transferring the ADP-ribosyl moiety from NAD^+^ to protein receptors, leading to depletion of ATP and subsequent cell death. This mechanism underlies cardiomyocyte apoptosis and necroptosis following I/R injury (Fiorillo et al., 2006; Lord et al., 2009).

When PARP1 is activated, PARylation triggers histone H1 dissociation from Forkhead box O3 a (FOXO3a)-target gene promoters, promoting FOXO3a nuclear accumulation and enhancing its binding affinity to these promoters, thereby upregulating autophagy-related gene expression. PARP1 mediates FOXO3a PARylation and facilitates its phosphorylation at residues T32, S252, and S314, which induces nuclear export of FOXO3a, suppresses its transcriptional activity and target gene expression, and ultimately drives myocardial hypertrophy (Lu et al., 2016). PARP1 activation further compromises mitochondrial metabolism by dysregulating FOXO3a-dependent autophagy, thereby accelerating cardiomyocyte death. PARP1 silencing or specific pharmacological inhibitors attenuate ischemia-induced FOXO3a hyperactivity, suppressing cardiomyocyte apoptosis, fibrotic remodeling, and pathological cardiac hypertrophy (Wang et al., 2018a).

PARP inhibition therapy mitigates mitochondrial ROS production and cardiac remodeling while restoring mitochondrial oxidative metabolism and left ventricular function (Ba & Garg, 2011). Pharmacological PARP inhibition also exerts cardioprotective effects across various cardiovascular injuries and heart failure-related cardiac/endothelial dysfunction by reducing plasma biomarkers of cardiomyocyte necrosis and downregulating inflammatory responses. Preserving cellular energy stores constitutes a key mechanism through which PARP inhibition protects against reperfusion injury, thereby improving myocardial functional recovery following cardiac arrest (Kaplan et al., 2005; Yamazaki et al., 2013). In cellular experiments, studies demonstrate that oxidative stress triggers apoptosis signal-regulating kinase 1 (ASK-1)-dependent activation of c-Jun N-terminal kinase (JNK), whereas PARP1 inhibition suppresses JNK activation (Bartha et al., 2009; Mester et al., 2009). Racz et al. (2010) confirmed that H_2_O_2_ treatment significantly increases p38 and JNK1/2 phosphorylation, while PJ-34—a pharmacological PARP inhibitor—reduces H_2_O_2_-induced p38 elevation and inhibits JNK activation below baseline levels in WRL-68 cells. Under basal conditions without H_2_O_2_, PJ-34 does not affect p38 but nearly abolishes JNK activation. Furthermore, PJ-34 treatment enhances cell viability following I/R injury, significantly reducing apoptosis while shifting cell death mechanisms from caspase-independent to caspase-dependent pathways. PARP1 inhibition attenuates oxidative stress-induced mitochondrial membrane potential collapse and secondary ROS generation (Du et al., 2003). In PJ-34-treated cardiomyocytes subjected to ischemia-reperfusion, oxidative stress and PARP1 activity decrease, NAD^+^/ATP depletion attenuates, and mitochondrial damage lessens. PJ-34 treatment not only enhances ischemic cell viability but also reduces apoptosis (Singh & Kang, 2011). Under TGF-β1 stimulation, both PARP1 expression and autophagy increase in cardiac fibroblasts (CFs). PARP1 inhibition partially blocks TGF-β1-induced CF activation by downregulating autophagy, thereby attenuating post-myocardial infarction fibrosis and improving cardiac function. PARP1 inhibition with 4-Aminobenzoic Acid Derivative (4-AB) alleviates myocardial fibrosis and autophagy in infarcted rats (Wang et al., 2018b), while another PARPi 4-Amino-1,8-naphthalimide (4-AN) significantly diminishes AIF-induced apoptosis and improves cardiac function (Singh & Kang, 2011).

Specificity protein 1 (SP1) may exert critical transcriptional regulation in myocardial infarction (MI), where PARP inhibition significantly reduces SP1 expression, ablates its positive regulatory effect on BCL2/adenovirus E1B 19 kDa-interacting protein 3-like (BNIP3L, also name as NIX) transcription, and concurrently suppresses cardiomyocyte viability (Geng et al., 2021). Research reveals that the phosphoinositide 3-kinase (PI3K)/Akt pathway modulates PARPi efficacy: PI3K blockade diminishes PARP inhibitor-mediated phosphorylation of Akt and GSK-3β during ischemia-reperfusion, substantially impairs recovery of ATP and creatine phosphate levels, and attenuates the protective effects of PARPi on infarct size reduction and cardiac functional recovery. This challenges the initial paradigm that NAD^+^ preservation and resultant ATP pool restoration constitute the exclusive mechanism underlying PARPi-mediated cyto-protection, demonstrating that PI3-kinase/Akt pathway activation and its downstream processes contribute equally, if not more substantially, to the cardio-protection afforded by PARPi during reperfusion injury (Kovacs et al., 2006).

In stroke and neurodegenerative diseases, Amelparib (JPI-289, a novel of PARPi) was found can reduce the infarct volume and brain swelling (Kim et al., 2018), by up-regulating regulatory T cells (Tregs) (Noh et al., 2018). For acute, life-threatening conditions such as stroke, myocardial infarction, circulatory shock, PARPi may interfere with NAD^+^-dependent enzymatic processes beyond PARP1. However, this off-target effect may clinically acceptable given the limited therapeutic alternatives and marginal efficacy of existing treatments for these critical diseases. Collectively, these findings indicate that selective pharmacological modulation of PARP1 represents a promising therapeutic strategy for mitigating ischemia-reperfusion injury.

PARP1 exerts dual and context-dependent functions in ischemia–reperfusion injury. While excessive activation promotes energy collapse, inflammation, and cell death, basal PARP1 activity can support adaptive signaling through FOXO3a, autophagy, and pro-survival pathways during myocardial remodeling. This mechanistic duality underscores the need to carefully delineate therapeutic timing and context for PARP inhibition, ensuring efficacy in acute ischemic events without perturbing essential metabolic or immune functions.

#### Cardiomyopathy and myocardial hypertrophy

PARP inhibition exerts no direct antihypertensive effect but mitigates hypertension-induced myocardial remodeling. Chronic PARP suppression induces persistent beneficial alterations in oxidative stress-associated signaling pathways (Bartha et al., 2009), conferring significant protection against the transition from hypertensive heart disease to heart failure (Bartha et al., 2009; Magyar et al., 2014). PARPi additionally modulate multiple signal transduction cascades: they activate pro-survival pathways (PI3K/Akt-1/GSK-3β, PKCε) that preserve mitochondrial integrity and enhance cellular viability during oxidative stress (Deres et al., 2014; Miyamoto, Murphy & Brown, 2009), while simultaneously suppressing the mitogen-activated protein kinase (MAPK) pathway, including c-Jun N-terminal kinase (JNK) and p38 MAPK, *via* MKP-1 upregulation (Deres et al., 2014; Jin et al., 2018; Racz et al., 2010). However, reduced MCP-1 expression may paradoxically increase JNK phosphorylation, activating mitochondrial fission and autophagy to impair energy production. PARP inhibition enhances activation of cytoprotective kinases protein kinase B (Akt) and protein kinase Cε (PKCε), while reducing activity of maladaptive remodeling kinases PKCα/β, PKCζ/λ, and PKCδ (Bartha et al., 2008). Through these mechanisms, PARPi attenuate cardiac interstitial fibrosis, left ventricular hypertrophy, and heart failure progression.

Studies have revealed that mitochondrial translocation of PARP1/PAR adversely affects POLG replisome-mediated mitochondrial DNA (mtDNA) maintenance, exacerbating mitochondrial dysfunction, oxidative stress, and cardiac remodeling in Chagas disease. PARP1 inhibitor therapy demonstrates therapeutic potential to preserve mitochondrial health and left ventricular function in chronic cardiomyopathy induced by myocardial ischemia and other etiologies (Ba & Garg, 2011). NAD^+^-dependent histone deacetylase Sirtuin 6 (SIRT6) inactivation during angiotensin II (AngII)-induced cardiac hypertrophy can be counteracted by PARP inhibition-mediated NAD^+^ replenishment, which reverses AngII-driven SIRT6 suppression. Restored SIRT6 activity attenuates myocardial hypertrophy *via* NF-κB signaling inhibition (Liu et al., 2014b).

In diabetic cardiomyopathy, although PARPi exhibits no significant effect on pre-existing hyperglycemia (Zakaria et al., 2016), PARPi elevates levels of SIRT1 and peroxisome proliferator-activated receptor gamma coactivator 1-alpha (PGC-1α), thereby attenuating oxidative stress, inflammation, and fibrotic remodeling (Waldman et al., 2018). PARP1 suppression in diabetic animal models protects cardiomyocytes from inflammatory responses and ROS overproduction (Waldman et al., 2018; Zakaria et al., 2016), while also preserving cardiac function and ameliorating metabolic dysregulation in diabetic hearts (Waldman et al., 2018).

Notably, doxorubicin (Dox)-induced cardiotoxicity—a major limitation in its clinical use—has been closely linked to PARP1 hyperactivation. PARP1 inhibitors effectively attenuate this toxicity, offering a promising strategy to enhance chemotherapeutic safety (Chen et al., 2025; Jiang et al., 2012). Pharmacological PARP inhibition protects cardiomyocytes against both chronic low-grade oxidative stress and acute severe oxidative damage *via* mitochondrial stabilization, positioning it as a promising therapeutic target for preventing and treating myocardial remodeling and heart failure (Ordog et al., 2021).

In conclusion, PARPi demonstrate substantial cardioprotective potential across multiple forms of cardiomyopathy and pathological myocardial remodeling, acting primarily through the regulation of oxidative stress, and mitochondrial homeostasis to mitigate the progression of hypertensive, ischemic, infectious, and metabolic cardiomyopathies.

Moreover, the central role of PARP1 in mitochondrial integrity, NAD^+^ metabolism, and its interaction with the Sirtuin family underscores the importance of precisely identifying patient populations and optimizing therapeutic timing. Although PARPi consistently confer protection in cardiac injury models such as DOX-induced cardiotoxicity, the multifaceted effects on immune and metabolic pathways present notable challenges for clinical translation. Future research should prioritize defining optimal dosing windows, stratifying interventions by disease stages, and integrating PARPi with complementary strategies, to fully realize the therapeutic potential of PARP-targeted therapies in cardiomyopathy and heart failure.

#### Atherosclerosis and inflammation

In atherosclerosis and inflammation, 3-aminobenzamide (3-AB), the first PARPi developed for cardiovascular indications—demonstrated a trend toward reducing plasma C-reactive protein and interleukin-6 levels without significantly lowering myocardial injury biomarkers in clinical studies (Pacher & Szabó, 2007, 2008). Mechanistically, PARPi decreases oxidative stress markers while reducing apoptosis in endothelial and smooth muscle cells (Levi & Stroes, 2008). Furthermore, PARPi diminishes both plaque volume and number, induces favorable structural modifications within plaques, and disrupts plaque progression. Vascular calcification is found to be a powerful and independent risk factor for cardiovascular and cerebrovascular mortality. Yang et al. (2025a) found PARylation level of DNA polymerase gamma were increased in calcified VSMCs from PARP1 activation, led to mitochondrial dysfunction and Adora2a (adenosine receptor A2A)/Rap1 (Ras-associated protein 1) signaling pathway activation to induce VSMC ferroptosis, which ultimately aggravated vascular calcification. PARPi could potentially serve as novel therapeutic strategies for vascular calcification (Yang et al., 2025a).

PARP1 inhibitors mitigate the severity of inflammatory processes across multiple organ systems (Gonzalez-Rey et al., 2007; Moroni, 2008; Mota et al., 2005; Scott et al., 2004; Soriano et al., 2002; Szabó, 2006; Tentori, Portarena & Graziani, 2002). In neurological contexts, these agents reduce cerebral infarction and neutrophil infiltration following transient focal cerebral ischemia (Couturier et al., 2003; Mosca et al., 2011; Tentori, Portarena & Graziani, 2002), confer protection against traumatic brain injury by suppressing nitric oxide overproduction (Wallis, Panizzon & Girard, 1996), alleviate motor deficits, and improve behavioral outcomes (Ding et al., 2001). Furthermore, PARP1 inhibition prevents neurobehavioral and neurochemical abnormalities (Mosca et al., 2011; Sriram et al., 2015), with accumulating evidence establishing their neuroprotective efficacy in diverse central nervous system pathologies (Besson et al., 2003; Jacewicz et al., 2009; Mosca et al., 2011; Nukuzuma et al., 2013). Pharmacologic inhibition of PARP1 by Olaparib rescued NAD^+^ levels and alleviated aging-associated blood–brain barrier (BBB) leakage, and the endothelial Connexin 43 (Cx43)-PARP1-NAD^+^ pathway can combat aging-associated BBB leakage with neuroprotective implications (Zhan et al., 2023).

PARP1 inhibition has been demonstrated to protect against endothelial dysfunction in shock, hypertension, and heart failure (Szabó, 2005; Zakaria et al., 2016). Sirtuins confer multifaceted endothelial protection, and PARP1 hyperactivation-induced endothelial injury involves NAD^+^ depletion that impairs the activity of Sirtuins. Research indicates that PARP inhibitor therapy reduces PARP1-mediated NAD^+^ consumption, suppresses mTOR phosphorylation (Qu et al., 2017), and elevates intracellular NAD^+^ bioavailability for sirtuins. This restores NAD^+^ levels in aged vasculature, enhancing Sirtuin-mediated endothelial protection (Alhosaini et al., 2021). Combined PARP1 inhibition and NAD^+^ augmentation synergistically harness both the anti-aging capacity of SIRT1 pathways and the anti-inflammatory effects mediated through reduced NF-κB activation (Alhosaini et al., 2021; Oliver et al., 1999). Given PARPi’s pivotal role in inflammation and cell death—processes essential for atherosclerotic plaque advancement and rupture—these findings indicate that PARP inhibition may therapeutically intercept these pathological cascades, positioning it as a promising strategy for atherosclerosis management (Oumouna-Benachour et al., 2007).

Beyond the established role of PARP1 in atherosclerosis, emerging evidence suggests that PARP9 and PARP14 also contribute to the pathogenesis of this disease. Iwata et al. (2016) demonstrated that PARP9 and PARP14 are closely associated with interferon-γ (IFNγ) signaling in macrophages. Specifically, PARP9 enhances IFNγ-induced responses, whereas PARP14 suppresses them, thereby establishing a functional balance that shapes vascular inflammation. One of the underlying mechanisms is that PARP9 inhibits PARP14-dependent mono-ADP-ribosylation of signal transducer and activator of transcription 1 (STAT1). The functional balance has important implications for vascular inflammation. Disruption of this equilibrium has significant implications for atherosclerosis (Iwata et al., 2016). Suppression of PARP14 activity promotes macrophage activation and accelerates plaque progression, while silencing PARP9 may alleviate macrophage-mediated vascular inflammation. Although there are currently no specific inhibitors of PARP9, several potent and selective small-molecule PARP14 inhibitors—such as RBN-3143 and Q22—have been developed, with some progressing into clinical trials for inflammatory diseases (Polasek et al., 2025; Wu et al., 2025). However, unlike in cancer or atopic dermatitis where PARP14 inhibition exerts anti-inflammatory effects, PARP14 appears to play a protective, anti-inflammatory role in atherosclerosis. This raises concern that PARP14 inhibition might inadvertently exacerbate vascular inflammation and plaque development. Consequently, the development of PARP9-specific inhibitors and PARP14-specific agonists, combined with advanced strategies for tissue-targeted drug delivery, represents a critical frontier for therapeutic innovation in atherosclerosis.

Impaired energy metabolism, oxidative stress, and dysregulated intracellular signaling pathways play pivotal roles in the pathogenesis of cerebrovascular and cardiovascular diseases. While PARPi exert profound modulatory effects on these processes, their clinical development for cerebrovascular/cardiovascular applications lags significantly behind oncology indications. This disparity stems from incomplete understanding of PARP-involved signaling networks: prior research has failed to delineate sufficiently robust pathways for targeted intervention. Collectively, these emerging lines of evidence underscore the substantial therapeutic potential of PARPi in the management of cardiovascular and cerebrovascular diseases. Beyond their well-established applications in oncology, PARP inhibition can modulate oxidative stress, preserve mitochondrial function, attenuate inflammatory signaling, and prevent energy collapse across diverse ischemic, hypertrophic, and inflammatory pathologies. These findings provide crucial mechanistic insight to guide the rational design of next-generation PARPi optimized for cardiovascular and cerebrovascular indications.

**Figure 3:**
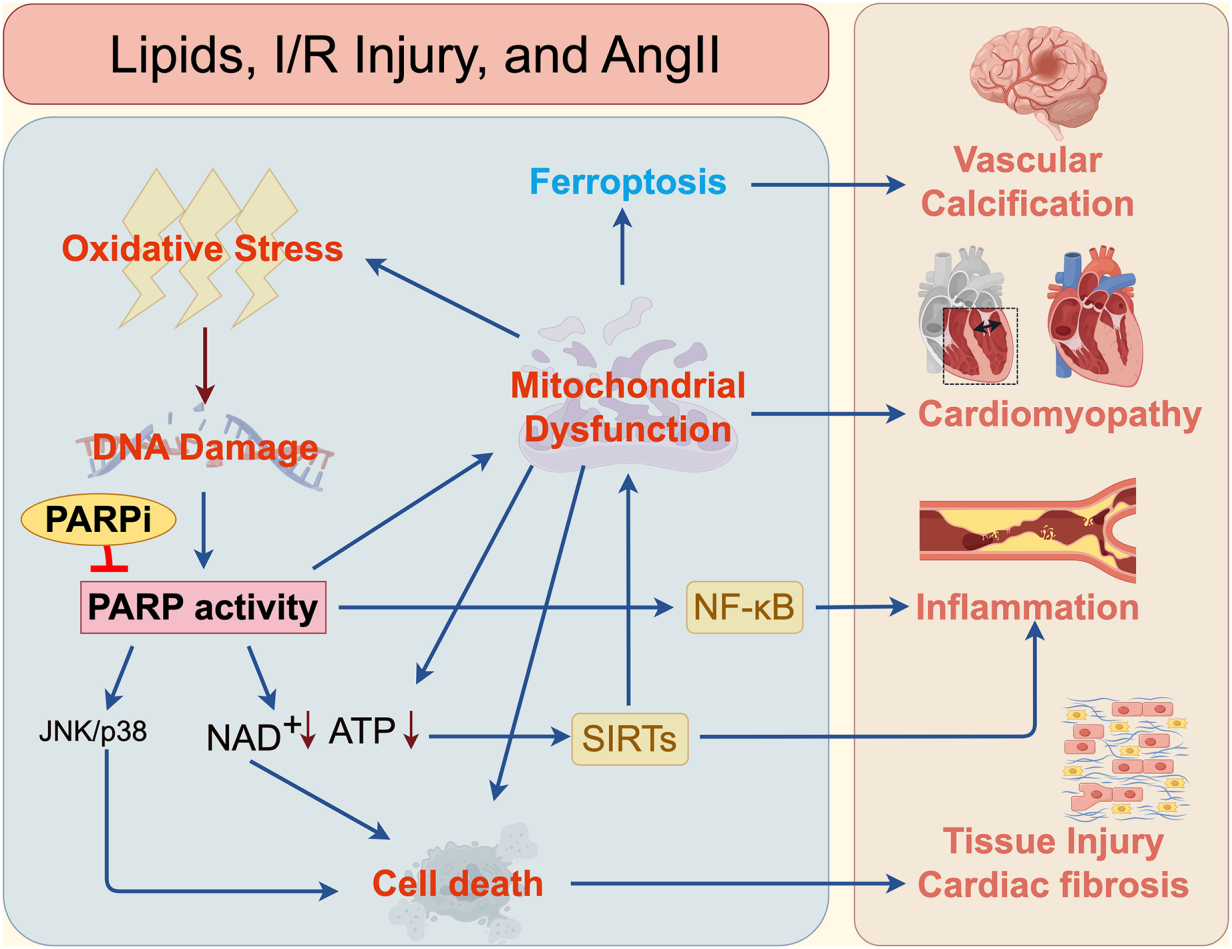
Mechanisms of PARPi in cardiovascular and cerebrovascular diseases. The mechanisms by which PARP and PARPi contribute to cardiovascular and cerebrovascular diseases, such as vascular calcification, cardiomyopathy, inflammation, atherosclerosis, and fibrosis.

As the field progresses, integrating these mechanistic insights with precision pharmacology, optimized dosing regimens, and disease stage–specific therapeutic windows will be essential for translating PARPi safely and effectively into clinical practice for cardiovascular and cerebrovascular disorders (Fig. 3).

## Parpi: current development and clinical application

The development of PARPi has progressed through several distinct generations, evolving from rudimentary tools into precision medicines. The first generation of inhibitors, emerging in the 1970s and 1980s, was exemplified by the nicotinamide analogue 3-AB. These agents enhanced the cytotoxicity of DNA-damaging agents by inhibiting PARP1’s catalytic activity, but were limited by low potency (up to 1,000-fold weaker than modern inhibitors) and poor selectivity, necessitating high concentrations that risked off-target effects on other cellular pathways. The second generation, developed in the 1990s, featured quinazoline-based analogues such as PD128763 and NU1025 (Calabrese et al., 2003). These compounds incorporated a carboxamide group that formed stable hydrogen bonds within the PARP1 catalytic site, resulting in a 50-fold increase in potency and improved selectivity over their predecessors.

A paradigm shift occurred with the advent of third-generation inhibitors, including Olaparib, Rucaparib, Niraparib, and *etc*. The enhanced cytotoxicity has led to significant clinical success, and currently, four PARPi—Olaparib, Rucaparib, Niraparib, and Talazoparib—are approved by the U.S. FDA for indications including ovarian and breast cancer. Concurrently, Pamiparib and Fluzoparib have been approved in China, and additional agents continues to advance through pre- or clinical stages. While the developmental trajectory of PARPi has been overwhelmingly driven by their application in oncology, their fundamental roles in modulating DNA repair, cell death, and inflammation have spurred investigation into their utility for non-malignant conditions. The preclinical findings suggest that PARPi may offer significant benefits to patients at risk of cardiovascular and cerebrovascular diseases. This section will review the clinical trial landscape for PARPi in the context of cardio- and cerebrovascular diseases (Table 1).

**Table 1:** Development and clinical application of PARPi in cardiovascular and cerebrovascular diseases.

Name of PARPi	Application (Cardiovascular and cerebrovascular diseases)	Status	Phase	Ref
Olaparib	Huntington’s disease	preClinical	–	Oumouna-Benachour et al. (2007)
	Transient cerebral ischemia	preClinical	–	Teng et al. (2016)
	Neurodegeneration	preClinical	–	Zhan et al. (2023)
	Dox-induced cardiotoxicity	preClinical	–	Chen et al. (2025)
Rucaparib	Dox-induced cardiotoxicity	preClinical	–	Ali et al. (2011)
Veliparib	Intracerebral hemorrhage	preClinical	–	Fu et al. (2024)
Amelparib	Acute Ischemic Stroke	NCT03062397	2	No results posted
MP-124	Acute Ischemic Stroke	preClinical	–	Matsuura et al. (2011)
3-AB	ST-Elevation Myocardial Infarction	NCT00271765	2	Morrow et al. (2009)
	Heart surgery	NCT00271167	2	No results posted

### Olaparib

As the first PARPi to gain FDA approval in 2014, Olaparib is a cornerstone of therapy for cancers with HRD. Olaparib has demonstrated robust efficacy and has been approved for the treatment of *BRCA1/2*-mutated breast, ovarian, prostate, and pancreatic cancers (Shah et al., 2025). Exploring its utility beyond cancer, Olaparib exhibit neuroprotection against excitotoxicity-induced cortical projection neuron death in Huntington’s disease and transient cerebral ischemia (Paldino et al., 2020; Teng et al., 2016). Olaparib additionally dilates constricted arteries in rats *via* its nicotinamide pharmacophore, conferring vasoactive effects (Senra et al., 2011). The extrapolation of Olaparib’s clinical utility beyond oncology warrants further exploration. Preclinical studies have also demonstrated that the Olaparib effectively ameliorates age-associated BBB impairment driven by Cx43 deficiency, indicating that PARPi may rescue endothelial cell senescence and the consequent increase in BBB permeability (Zhan et al., 2023).

### Rucaparib

Rucaparib was approved on an accelerated basis by FDA in 2016, for patients with advanced ovarian cancer related to deleterious *BRCA1/2* mutations, after they had been treated with two or more chemotherapy courses (Ledermann et al., 2025). Rucaparib demonstrates efficacy both as monotherapy and in combination with standard chemotherapeutics (Plummer et al., 2008). It specifically suppresses DNA damage-induced NF-κB activity—a stress-responsive transcription factor regulating apoptosis, proliferation, angiogenesis, and metastasis—thereby blocking NF-κB-mediated anti-apoptotic gene expression (Barkett & Gilmore, 1999; Wang et al., 1998). Rucaparib has demonstrated potential in cardiovascular protection by mitigating Dox-induced cardiotoxicity without altering its pharmacokinetics, and inhibits myosin light-chain kinase (MLCK) activity, a key mediator of muscle contraction (Ali et al., 2011).

### Niraparib

Niraparib, the selective oral PARP1/2 inhibitor approved by FDA in 2017, characterized by its indazole carboxamide scaffold, is approved for treating ovarian, fallopian tube, and primary peritoneal cancers, with ongoing investigations extending to other malignancies including brain tumors (Martins et al., 2021). Niraparib demonstrates efficacy in both *BRCA1/2*-mutated and wild-type tumors, securing approval as second-line maintenance therapy for all ovarian cancer patients regardless of *BRCA* status (Booth et al., 2018).

### Talazoparib

Talazoparib is distinguished as the most potent PARP trapping agent among clinically available PARPi clinically used for the therapy of breast cancer, and was approved by FDA in 2018 (Murai et al., 2012, 2014). Talazoparib also has shown antitumor activity against different cancer types, including non-small cell lung cancer (Sabet et al., 2022).

### Veliparib

Veliparib is a PARP1/2 inhibitor primarily characterized by its exceptional ability to cross the blood-brain barrier. Currently, Veliparib is in multiple clinical trial phases, including studies for ovarian cancer and glioblastoma, among others (Coleman et al., 2025; Karajannis et al., 2025). Veliparib demonstrates neuroprotective effects *in vitro* against excitotoxicity-induced cortical neuron death (Xu et al., 2016) and *in vivo* following transient cerebral ischemia (Teng et al., 2016). In intracerebral hemorrhage (ICH) models, it suppresses glial activation and downregulates pro-inflammatory cytokine production. Veliparib reduces microglial counts, inhibits peripheral immune cell infiltration into the brain, and preserves blood-brain barrier integrity (Fu et al., 2024). Through these anti-inflammatory mechanisms, veliparib improves neurological outcomes, representing a promising novel therapeutic approach for ICH.

### Preclinical inhibitors

3-Aminobenzamide (3-AB) is the first prototypical PARPi that competitively blocks the catalytic domain of PARP1/2. Preclinically, 3-AB demonstrates radio- and chemo-sensitizing effects in tumor by exacerbating DNA damage accumulation. Phase 2 clinical trials demonstrated that 3-AB was well-tolerated and exhibited a trend toward inflammation suppression in patients with ST-elevation myocardial infarction (MI) (Morrow et al., 2009). As the first-generation PARPi, its low potency and poor pharmacokinetics limit clinical translation, having been superseded by high-affinity PARPi in therapeutic applications. However, 3-AB yet holds seminal academic significance (Chiu et al., 2012).

PJ34 inhibits PARP1 activity (Ratnam & Low, 2007; Zhang et al., 2024), as a competitive NAD^+^ substrate analog. Beyond oncology, PJ34 prevents endothelial dysfunction and hypertension in rats (Walsh et al., 2012), mitigates vascular inflammation and cerebral edema while improving reperfusion and vascular protection post-stroke (Black et al., 2006; El Amki et al., 2018; Nasrabady et al., 2012), and reduces VEGF-induced proliferation, migration, and tube formation in human umbilical vein endothelial cells (HUVECs) (Rajesh et al., 2006). PJ34 diminishes leukocyte infiltration, promotes structural and functional endothelial recovery, suppresses neointima formation (Zhang, Yang & Jennings, 2004), alleviates acute cardiac transplant rejection (Chen et al., 2021), and inhibits inflammatory conditions including colitis, autoimmune diabetes, and uveitis (Black et al., 2006; Hamby et al., 2007; Noël et al., 2006). This multifaceted activity positions PJ34 as a candidate for cardiovascular and inflammatory therapies, with targeted DNA repair inhibition representing a viable strategy against restenosis. However, current research remains largely confined to preclinical studies, and its clinical translation remains investigational.

MP-124, a novel PARPi, significantly ameliorated not only the neurological deficits but also the infarct volume in monkey subjected to middle cerebral artery occlusion (Matsuura et al., 2011; Yamamura et al., 2015).

Notably, a groundbreaking study identified TRIP12, an E3 ubiquitin ligase, as a negative regulator of PARPi sensitivity. TRIP12 governs PARP1 homeostasis by promoting its proteasomal degradation, thereby constraining cytotoxic PARP-DNA complex accumulation. TRIP12 dysfunction elevates PARP1 levels and potentiates PARP trapping, creating a hyper-susceptible state to PARP inhibition (Gatti et al., 2020). This mechanistic insight opens novel therapeutic avenues for both chemo-sensitization and resistance reversal. Further expanding therapeutic applications, evolving combination strategies will amplify the clinical impact of PARPi, ultimately enhancing patient survival and quality of life. Additionally, next-generation PARPi—including selective PARP1 antagonists and PARP degraders (such as proteolysis-targeting chimeras, (PROTACs))—herald transformative approaches for malignancies and non-oncological conditions, offering novel and effective therapeutic avenues for diverse patient populations.

Collectively, these mechanistic insights highlight the broad therapeutic potential of PARPi. As our understanding of PARP biology continues to expand beyond oncology, elucidating the functional diversity and pharmacological evolution of different PARPi will be critical for guiding their rational application in emerging therapeutic domains such as cardiovascular and cerebrovascular diseases.

## The challenge for clinical translation

### Therapy resistance

The significant therapeutic benefits demonstrated by PARPi across diverse animal models of human diseases underscore their potential for clinical translation. However, critical safety challenges must be resolved before these agents can be effectively deployed for chronic human conditions. Foremost among current clinical limitations is acquired resistance (Noordermeer & van Attikum, 2019), with widespread PARP inhibitor use likely accelerating resistance development. Despite the therapeutic promise of PARPi in both monotherapy and combination regimens, their clinical utility faces significant constraints. A primary challenge is the inevitable emergence of acquired resistance following prolonged administration. Although these agents specifically target HR-deficient tumors, resistance mechanisms frequently evolve. Intriguingly, somatic *PARP1* mutations identified in resistant tumors abrogate PARP trapping—the formation of cytotoxic PARP1-DNA complexes—thereby conferring therapeutic escape (Segalés et al., 2025; Zhang et al., 2023). Genetic alterations like mutations, gene amplifications, and deletions have long been considered the principal drivers of drug resistance. However, an expanding body of evidence now underscores the critical contribution of non-genetic mechanisms, including phenotypic plasticity, cellular reprogramming, and epigenetic dysregulation (Maggiorani et al., 2024). To counteract this, strategies are being developed to co-target multiple vulnerabilities through either polygenic synthetic lethality or synergistic combination therapies, thereby disabling compensatory signaling. These innovative approaches are still nascent, demanding rigorous investigation to definitively establish their efficacy, safety, and optimal clinical applications.

### Adverse event and toxicity

Moreover, the on-target toxicity of PARPi poses a critical limitation. Chronic high-dose exposure induces PARP-DNA complex accumulation even in proliferating normal tissues (*e.g*., bone marrow, intestinal epithelium), triggering unintended cell death through replication stress. Most clinically developed PARPi competitively bind to the NAD^+^ site on PARP1 (Murai et al., 2012). All current clinical and preclinical PARP1 inhibitors are NAD^+^-competitive mimetics featuring a lactam ring or primary amide conformationally locked *via* intramolecular hydrogen bonding with adjacent heteroatoms (Papeo et al., 2019). This design strategy has yielded NAD^+^-like PARPi that inadvertently target not only PARP but also other NAD^+^/nucleotide-dependent enzymatic pathways. Most inhibitors in clinical trials exhibit additional mechanisms—such as inducing cytotoxic PARP-DNA complex formation or altering phosphorylation signaling pathways—that may compromise normal cellular functions (Dal Piaz et al., 2015). Existing inhibitors like Olaparib carry significant adverse effects, particularly hematological toxicity, during chronic administration (Nilov et al., 2020). Chronic PARP inhibition may elevate mutation rates and carcinogenic risk. Moreover, as several PARP family members have undefined functions, non-selective suppression of PARylation could provoke severe unforeseen side effects. Therefore, when evaluating the risk-benefit ratio of PARPi for therapeutic applications, a clear distinction must be made between acute *vs*. chronic treatment protocols and life-threatening *vs*. non-critical disease indications. A meta-analysis research indicated that treatment with PARPi confers an increased risk of developing myelodysplastic syndrome (MDS) and acute myeloid leukemia (AML) relative to placebo (Morice et al., 2021). A large-scale observational study in the England demonstrated a higher relative risk of MDS or AML incidence in patients receiving PARPi maintenance therapy than in those treated with chemotherapy alone, although the absolute risk in both groups was minimal (Steventon et al., 2025). Consequently, while the absolute incidence of secondary MDS/AML remains low among patients on PARPi maintenance, a high degree of clinical vigilance is warranted for the early detection of these hematologic malignancies in at-risk patient populations.

### Scarcity of predictive biomarkers

A critical challenge in clinical application of PARP1 inhibitors lies in the scarcity of predictive biomarkers beyond *BRCA1/2* mutations. Recent studies reveal that acquired secondary mutations can restore *BRCA* functionality and HR proficiency in *BRCA*-defective tumors, while PARP1 downregulation in tumor subclones further diminishes synthetic lethality (Domagala et al., 2011; Lee et al., 2009; Skehan et al., 1990). For instance, only ~10% of pancreatic cancer patients with HRD (Golan et al., 2019), severely limiting therapeutic applicability. Although PARP1 expression and enzymatic activity have been proposed as predictive tools, significant discordance exists between PARP1 mRNA/protein levels and actual PARylation activity, nor do these parameters reliably predict PARP trapping efficacy. Emerging evidence suggests that pre-existing high PAR levels in tumors may serve as a superior biomarker over *BRCA* mutational status alone (Gottipati et al., 2010). Multimodal biomarker integration is anticipated to refine patient stratification in the coming decade (Oplustilova et al., 2012), combining *BRCA1/2* mutations, MRE11-RAD50-NBS1 (MRN) complex status, PARylation dynamics, Rad51 foci formation, and 53BP1 shuttling patterns.

PARPi are distinguished by their dual therapeutic utility, conferring both potent antitumor activity and significant cardio- and cerebro-protection. The advent of next-generation agents, such as AZD5305 (Herencia-Ropero et al., 2024) and AZD9574 (Johannes et al., 2024), has created unprecedented opportunities for PARP inhibition as a therapeutic strategy in cardiovascular and cerebrovascular medicine. A critical next step is the clinical translation of these preclinical findings to validate the cardioprotective attributes observed in animal models. The absence of comprehensive data on clinically approved PARPi in cardiac disease models necessitates additional research to explore their selectivity, pharmacokinetics, and relevance in cardiovascular and cerebrovascular scenarios.

## Conclusion

Despite major advances in understanding of PARP biology, significant knowledge gaps remain in elucidating how PARP1 and other PARP family members integrate DNA repair, metabolic regulation, inflammation, and cell death across different pathological contexts. Current evidence indicates that while PARPi have achieved remarkable success in oncology through mechanisms such as synthetic lethality and PARP–DNA trapping, their effects in cardiovascular and cerebrovascular diseases differ fundamentally due to the low proliferative capacity and high metabolic vulnerability of cardiac and nerve cells.

Preclinical studies have demonstrated substantial therapeutic potential of PARPi in these settings, including preservation of mitochondrial function, attenuation of oxidative stress, modulation of inflammatory signaling, and protection against I/R injury. However, critical challenges persist, particularly in defining disease-specific therapeutic windows, understanding long-term metabolic consequences, characterizing off-target enzymatic effects, and clarifying the distinct roles of individual PARP isoforms in cardiac and neural pathologies.

Moving forward, research efforts should prioritize the development of isoform-selective PARPi, PARP agonists, PROTAC-based PARP degraders, and targeted delivery systems tailored for cardiovascular and cerebrovascular indications. Combining PARPi therapy with complementary strategies—such as NAD^+^ replenishment, mitochondrial stabilization, and anti-inflammatory treatments—may further enhance efficacy while minimizing toxicity. Additionally, high-resolution single-cell and spatial transcriptomics will be indispensable for delineating cell type–specific PARP functions in diseased cardiac and neural tissues.

In conclusion, PARPi have revolutionized cancer therapy through their foundation in DNA damage response (DDR) biology, yet their full potential in cardiometabolic and neurovascular diseases remains largely unexplored. Strategic innovation, guided by mechanistic insight and precision medicine, holds the promise of expanding next-generation PARPi into transformative therapeutics beyond oncology.

## Abbreviations

AIF: apoptosis-inducing factor
AMPK: AMP-activated protein kinase
ATP: adenosine triphosphate
BBB: blood–brain barrier
BRCA1/2: breast cancer susceptibility gene 1/2
DDR: DNA damage response
DSB: double-strand break
FOXO3a: Forkhead box O3
HIF-1α: hypoxia-inducible factor-1 alpha
HR: homologous recombination
HRD: homologous recombination deficiency
I/R: ischemia–reperfusion
JNK: c-Jun N-terminal kinase
MI: myocardial infarction
NAD^+^: nicotinamide adenine dinucleotide
NF-κB: nuclear factor-κB
NHEJ: non-homologous end joining
PARP: poly(ADP-ribose) polymerase
PARPi: PARP inhibitor(s)
PARylation: poly(ADP-ribosyl)ation
PI3K: phosphoinositide 3-kinase
ROS: reactive oxygen species
SSB: single-strand break

## Additional Information and Declarations

### Data Availability

The following information was supplied regarding data availability:

This is a literature review.
